# Re-optimizing the Time Frame for Classifying Cerebral Venous Sinus Thrombosis: An Unmet Need

**DOI:** 10.7759/cureus.75951

**Published:** 2024-12-18

**Authors:** Nikhil Vojjala, Supriya Peshin, Jayalekshmi Jayakumar, Nikhil Kotla, Adit Dharia, Mamtha Balla, Geetha Krishnamoorthy

**Affiliations:** 1 Internal Medicine, Trinity Health Oakland Hospital, Pontiac, USA; 2 Internal Medicine, Norton Community Hospital, Norton, USA; 3 Internal Medicine, The Brooklyn Hospital Center, New York, USA; 4 Internal Medicine, HCA Florida Oak Hill Hospital, Florida, USA; 5 Infectious Disease and Transplant, MD Anderson Cancer Center, Houston, USA

**Keywords:** cerebral venous sinus thrombosis (cvst), cerebral venous thrombosis cvt, covid 19, iscvt2, re-spect

## Abstract

Cerebral venous thrombosis (CVT) is a rare and complex form of stroke, representing a small percentage of all stroke cases. The disease's clinical presentation is highly variable, involving a wide range of medical specialists due to its diverse manifestations. Over the past decade, significant advancements in understanding CVT have been made, particularly in light of the COVID-19 pandemic and subsequent vaccination efforts. Current classification systems of CVT, which categorize the disease as acute, subacute, or chronic based on symptom onset, vary significantly in their criteria and practical relevance. Key challenges include aligning these time-based classifications with real-world delays in diagnosis and treatment, as well as understanding the timing and nature of complications such as raised intracranial pressure and the need for decompressive procedures. Radiological studies of clot morphology provide additional insights, suggesting that clot characteristics may indicate the recanalization potential and influence treatment strategies. However, the relationship between clot recanalization and clinical outcomes remains debated. A comprehensive classification that integrates clinical, radiological, and prognostic parameters could aid in better managing CVT and improving patient outcomes.

## Introduction and background

Cerebral venous thrombosis (CVT) is a rare and complex condition with a highly diverse clinical presentation and a multitude of underlying causes. Over the past decade, significant advancements have been made in understanding cerebral venous strokes, driven in part by intensified research efforts during the global COVID-19 pandemic and the associated vaccination campaigns. CVT accounts for approximately 0.5-1% of all strokes and presents a unique diagnostic and therapeutic challenge across a wide range of medical specialties [1]. This condition is not confined to neurologists but frequently requires the involvement of emergency medical practitioners, neurosurgeons, rheumatologists, hematologists, and neuro-ophthalmologists due to its multifaceted clinical spectrum. Despite substantial data on its diagnosis, management, and prognostic factors, there remains a notable gap in achieving a universal consensus on a standardized classification system for patients with cerebral venous sinus thrombosis. Such a framework is essential to streamline diagnosis, guide treatment, and predict outcomes effectively across diverse clinical settings.

## Review

The earliest documented description of cerebral venous thrombosis (CVT) can be traced back to 1825 in French literature, where Ribes detailed the case of a 45-year-old man who succumbed after enduring a month-long history of severe headaches, epileptic seizures, and delirium [2]. History dates back to 1893 when Quinke et al. first introduced the term “meningitis serosa” for patients with chronic headaches with no positive signs on imaging [3]. However, the entity underwent numerous changes in nosology, and it was way back in 1952 that the term was replaced with "benign intracranial hypertension". This term was widely accepted for many decades until its "not so benign" nature, causing significant visual impairment, was recognized. As a result, it was renamed "idiopathic intracranial hypertension" (IIH). While IIH is a suitable term, the frequent overuse and misuse of the phrase “IIH without papilledema,” combined with the need for better awareness of radiological findings, clearer descriptions of the condition in children, and a more accurate understanding of normal cerebrospinal fluid (CSF) opening pressure in this age group, underscores the importance of revising its nomenclature within the medical community [4]. In late 2013, Friedman et al. suggested that while a subset of patients initially appeared to have no identifiable cause, some were later found to have treatable underlying conditions [5]. These cases were not adequately represented by the term “IIH” and required etiology-specific management protocols. This led to the realization that the term “IIH with secondary cause” was contradictory, prompting the development of the term “pseudotumor cerebri syndromes" (PTCS), which has since gained international recognition in the field of neurology.

Since then, CVT has been regarded as a secondary cause of IIH in PTCS [5]. Even though there is robust data on diagnosis, management, and prognostic factors in this disease, data, and the universal consensus are lacking in the classification schema of patients with cerebral venous sinus thrombosis. An ideal classification system for a particular disease should aim to create homogenous sub-groups considering their clinical characteristics, management, and long-term prognosis. Such basic defining criteria are missing in the schema for classifying CVT patients into acute, subacute, and chronic. Because of the heterogeneity existing in the classification of CVT across the world, we aimed to review the cut-offs used by various previous studies, describe their advantages and disadvantages, and finally propose our classification, considering the above basic characteristics. In the largest cohort of a prospective study of 624 patients, the international study on cerebral venous and dural sinus thrombosis (ISCVT cohort) classified patients into acute, subacute, and chronic CVT based on the time frame of first symptom onset. Acute being within 48 hours, subacute from 48 hours to 4 weeks, and chronic if more than four weeks [6]. Following this, several other cohorts, including the French cohort, the guideline framework of the American Heart Association, made the arbitrary cut-off of 48 hours for acute CVST [1,7,8]. However, several issues warrant further discussion. The median delay of presentation to the health care setting was four days, and the median duration for diagnosis of CVT was seven days in the ISCVT cohort. The median length of hospital stay in patients of CVT was 10 days in the French CVT cohort compared to the 17 days of the ISCVT cohort [6,7]. The cut-off of 48 hours for acute CVT does not satisfy the delay in diagnosis of seven days nor the median hospital stay. Indeed, a close look up at the timing of complications of raised intracranial pressure requiring decongestant therapy for relieving cytotoxic edema and in continuation requirement of decompressive surgical procedures were more pronounced with a median of four days after the onset of symptoms, as demonstrated in the ISCVT2 systematic review of 31 cases [9]. In an analysis of 13 patients who underwent decompressive craniotomy at our tertiary teaching center in Northern India, the timing of decompression after presentation to emergency ranged from seven hours to days after hospital admission [10].

It is also reported that patients who had delayed presentation of more than seven days of symptom onset were noted to have neuro-ophthalmological features in the form of progressive vision loss, higher grade of papilledema, and, if not intervened, leading to secondary optic atrophy [11]. Hence, early institution of intracranial pressure lowering therapies (medical management using carbonic anhydrase inhibitors and surgical management like subjecting to theco-peritoneal shunt, ventriculoperitoneal shunt, and optic nerve sheath fenestration) may prevent from having debilitating vision loss and thereby prevent visual morbidity. Though performing lumbar puncture is not shown to increase the progression of venous sinus thrombosis in the ISCVT cohort, the theoretical risk of thrombus progression due to local hemoconcentration cannot be completely excluded [12]. The optimal timing for safely performing a lumbar puncture (LP) in patients with cerebral venous sinus thrombosis (CVT) remains uncertain and is an area of ongoing research. LP transitions from being a relative contraindication during the acute phase to a preferred intervention in the subacute and chronic phases of CVT, particularly in cases with secondary chronic intracranial pressure (ICP) and progressive vision loss [13]. Radiologically, the luminal clot's morphological characteristics on various MRI sequences help determine its state. A clot is considered acute within the first five days after symptom onset, subacute between 5 to 15 days, and chronic if it persists beyond 15 days from symptom onset [14]. The inclusion of radiological characteristics, including the clot phase and morphology, is important as it may reflect the recanalization status achieved at the end of anticoagulation. Indeed, a meta-analysis of 694 patients revealed that higher recanalization rates were associated with a significant increase in favorable outcomes [15]. However, other recent studies, like the prospective RE-SPECT CVT trial (recanalization after cerebral venous thrombosis), did not reveal any relationship between recanalization status and functional outcome [16]. Secondly, this also has an impact on the treatment decision to be taken, such as performing the endovascular treatment. The success of endovascular therapy was observed when the thrombus was in the acute phase (within 10-14 days), as observed in various studies performed on deep venous thrombosis in the lower limb [17].

Additionally, neuro-ophthalmological symptoms form core across the spectrum of CVT patients [13,18]. In a study of 53 patients, diplopia and papilledema were found to be prominent in chronic CVT due to its slow onset and progression. This delay in diagnosing CVT often has a significant impact on vision [18]. Similarly, Eliseeva et al. studied 49 CVT patients across its spectrum: acute (within 2 weeks), subacute (2-4 weeks), and chronic (>4 weeks). They observed that while papilledema was a universal finding in all patients, vision loss was generally absent in acute and subacute CVT, except in cases with macular hemorrhages. However, in chronic CVT, because of persistent papillodema and post-papillodema, secondary optic atrophy vision loss is more prominent [13]. The cut-off time for this transition from a reversible asymptomatic visual impairment to progressive, debilitating vision loss is one of the important deciding factors in the overall outcome achieved in patients of CVT. Unfortunately, this area remains an enigma and an active area of research. The heterogeneity of classification systems available in the current literature is reviewed in Table 1.

**Table 1:** Heterogeneity of classification systems in the available literature in the sub-classification of patients with CVT.

Study	Type of study	Number of patients	Acute	Subacute	Chronic
Ferro JM et al. [6]	Prospective	624	2 days	2 days-28 days	>28 days
Bagan et al. [7]	Prospective	231	2 days	2 days-28 days	>28 days
Saposnik et al. [1]	Guideline	-	2 days	2 days-28 days	>28 days
Petrovic J et al. [8]	Retrospective	49	2 days	2 days-28 days	>28 days
Lian et al. [19]	Prospective	306	Within 14 days	Within 14 days	>14 days
Idiculla et al. [20]	Review	-	2 days	2 days- 28 days	>28 days
Yadegari et al. [18]	Prospective	53	2 days	2 days-14 days	>14 days
Wasay et al. [14]	Review of MRI features	-	5 days	5-15 days	>15 days
Eliseeva et al. [13]	Prospective study	49	14 days	14-28 days	>28 days

**Figure 1:**
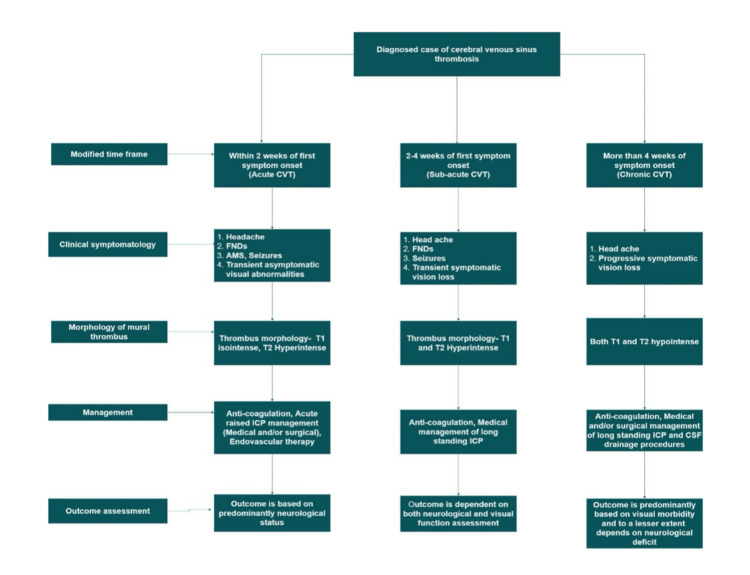
Pipeline depicting the homogeneity within the sub-group and heterogeneity between the subgroups of CVT patients. Image credits: authors. CVT: cerebral venous thrombosis, FNDs: focal neurological deficits, AMS: altered mental sensorium, ICP: intracranial pressure, CSF: cerebrospinal fluid.

Building on clinical parameters, radiological findings, outcome measures, and the natural progression of the disease, our proposed schema aims to establish clear cut-offs for categorizing patients with cerebral venous sinus thrombosis into acute, subacute, and chronic phases, as illustrated (Figure 1). 

## Conclusions

In conclusion, CVT remains an enigmatic condition characterized by its intricate presentations, diverse etiologies, and persistent challenges in classification. While significant progress has been made in understanding its clinical and radiological characteristics, the lack of a universally accepted classification system continues to create barriers to consistent diagnosis and treatment. The interplay between clinical manifestations, such as neuro-ophthalmological sequelae, and evolving radiological features underscores the necessity for a holistic approach that aligns timely interventions with long-term outcomes. Emerging evidence indicates the need for a more refined framework incorporating clinical phases, clot morphology, and tailored management strategies. By addressing these complexities and fostering consistency in care, a unified classification system promises to transform CVT management, improve patient prognosis, and mitigate preventable complications.
